# RPE65 variant p.(E519K) causes a novel dominant adult-onset maculopathy in 83 affected individuals

**DOI:** 10.21203/rs.3.rs-5849564/v2

**Published:** 2025-05-05

**Authors:** Eline Van Vooren, Filip Van den Broeck, Quinten Mahieu, Eline Geens, Mattias Van Heetvelde, Marieke De Bruyne, Stijn Van de Sompele, Sheetal Uppal, Eugenia Poliakov, Claire-Marie Dhaenens, Cheryl Y. Gregory-Evans, Lies Hoefsloot, Adriana Iglesias Gonzalez, Susanne Kohl, Theresia Zuleger, Tanguy Demaret, Dominant RPE65-p.(E519K) Consortium, Sari Tuupanen, Joke Ruys, Luc Van Os, Elise Platteau, Julie Jacob, Sascha Vermeer, Laurence Postelmans, Karin Dahan, Isabelle Maystadt, Florence Rasquin, Alberta A.H.J. Thiadens, Kirk A.J. Stephenson, Narin Sheri, Vasily Smirnov, Ian M. MacDonald, Kevin Gregory-Evans, T. Michael Redmond, Julie De Zaeytijd, Bart P. Leroy, Miriam Bauwens, Elfride De Baere

**Affiliations:** 1 Department of Biomolecular Medicine, Ghent University, Ghent, Belgium; 2 Center for Medical Genetics, Ghent University Hospital, Ghent, 9000, Belgium; 3 Department of Head and Skin, Ghent University, Ghent, 9000, Belgium; 4 Department of Ophthalmology, Ghent University Hospital, Ghent, 9000, Belgium; 5 Laboratory of Retinal Cell and Molecular Biology, National Eye Institute, NIH, Bethesda, 20892, United States; 6 Univ. Lille, Inserm, CHU Lille, U1172-LilNCog-Lille Neuroscience & Cognition, F-59000 Lille, 59000, France; 7 University of British Columbia, Department of Ophthalmology & Visual Sciences, Vancouver, BC, V6T 1Z4, Canada; 8 Department of Clinical Genetics, Erasmus Medical Centre, Rotterdam, 3015, The Netherlands; 9 Institute for Ophthalmic Research, Centre for Ophthalmology, University Hospital Tübingen, 72074, Germany;; 10 Institute of Medical Genetics and Applied Genomics, University Hospital Tübingen, 72074, Germany; 11 Centre de Génétique Humaine, Institut de Pathologie et de Génétique, Gosselies, 6041, Belgium; 12 Blueprint Genetics, Espoo, 02150, Finland; 13 Department of Ophthalmology, Vitaz, Sint-Niklaas, 9100, Belgium; 14 Department of Ophthalmology, Antwerp University Hospital, Antwerp, 2650, Belgium; 15 Department Ophthalmology, Maria Middelares Hospital, Ghent, 9000, Belgium; 16 Department of Ophthalmology, UZ Leuven, Leuven, 3000, Belgium; 17 Center for Human Genetics, UZ Leuven, Leuven, 3000, Belgium; 18 Department of Ophthalmology, CHU Brugmann, Brussels, 1020, Belgium; 19 Department of ophthalmology, Erasme Hospital, Université Libre de Bruxelles, Brussels, 1070, Belgium; 20 Department of Ophthalmology, Erasmus Medical Center, Rotterdam, 3015, The Netherlands; 21 Department of Ophthalmology and Visual Sciences, University of Alberta, Edmonton, T6G 2R3, Canada; 22 Sorbonne Université, INSERM, CNRS, Institut de la Vision, Paris, 75012, France; 23 Division of Ophthalmology and Center for Cellular and Molecular Therapeutics, Children’s Hospital of Philadelphia, Philadelphia, PA, 19104, United States

**Keywords:** Autosomal dominant, European ancestry, Founder, Inherited Retinal Disease (IRD), RPE65

## Abstract

Recessive RPE65-related retinopathy is an inherited retinal disease (IRD) that is a well-established target for gene therapy. Dominant RPE65-related retinopathy, however, due to Irish founder variant p.(D477G), is extremely rare. Here, we report the discovery, replication and characterization of a novel dominant retinopathy caused by RPE65 variant p.(E519K), identified in 83 individuals of European ancestry across IRD registries (Belgian discovery cohort, n=2,873; replication cohort, n=18,796). Long-read sequencing-based haplotyping revealed a shared region of 464 kb, supporting a founder effect. Genotype-phenotype data support dominant inheritance and phenotypic variability respectively, characterized by late-onset macular dystrophy with two main subtypes, a pathognomonic mottled subtype and a pattern dystrophy subtype. Functional studies showed that the p.(E519K) variant affects RPE65 enzymatic activity, correlating with lower protein expression. Protein modelling and cellular thermal shift assays further supported a destabilizing effect on protein structure. Overall, our work provides strong genetic, clinical, molecular and functional evidence for a novel dominant RPE65 retinopathy in multiple families in Europe and North America due to a Belgian founder variant. This discovery reduces the diagnostic gap in dominant IRD, particularly in individuals of European ancestry. Finally, it lays the foundation for developing therapeutic strategies targeting dominant RPE65 retinopathy.

## Introduction

Approximately two million people worldwide are affected by inherited retinal diseases (IRD), a clinically and genetically heterogeneous group of disorders leading to progressive or stable vision loss^1,2^. Over 290 genes have been implicated in IRD, one of which is *RPE65* (retinal pigment epithelium-specific 65 kDa), encoding a key enzyme in the visual cycle, which catalyzes the isomerization of all-*trans* retinyl ester to 11-*cis* retinol^3–5^. Biallelic pathogenic variants in the *RPE65* gene (OMIM*180069) are implicated in different forms of autosomal recessive (AR) IRD, including Leber congenital amaurosis type 2, early-onset severe retinal dystrophy, and retinitis pigmentosa. Patients with AR *RPE65*-IRD manifest a generalized phenotype with congenital or early-onset night blindness, progressive loss of visual field and loss of central vision^6^.

*Voretigene neparvovec-rzyl* (Luxturna^®^), the first gene therapy for IRD approved by FDA and EMA in 2017 and 2018 respectively, is a gene augmentation therapy involving subretinal injection of adeno-associated virus type 2 vectors containing wild type (WT) *RPE65* complementary DNA (cDNA), resulting in improved light sensitivity, visual field and navigational abilities^7–10^. Eligibility depends on the presence of biallelic (likely) pathogenic *RPE65* variants and sufficient viable outer retinal cells, emphasizing the importance of an early molecular diagnosis.

Autosomal dominant (AD) *RPE65*-IRD has been described only once and is due to the Irish founder variant c.1430A>G, p.(Asp477Gly), also known as p.(D477G). Non-penetrance and large phenotypic variability, ranging from foveal vitelliform lesions to extensive chorioretinal atrophy mimicking choroideremia, have been described in a few families worldwide^11–14^. In p.(D477G)-IRD knock-in mouse models only minimal phenotypic manifestations were observed^15–17^. Possible mechanisms of action involve aberrant RNA splicing and a toxic and/or dominant-negative effect through abnormal aggregate formation^15,17^.

Here, we describe the discovery, replication, clinical, molecular and functional characterization of a novel AD *RPE65*-related retinopathy in multiple families in Europe and North America due to a Belgian founder variant c.1555G>A, p.(Glu519Lys), hereafter referred to as p.(E519K). Our findings reduce the diagnostic gap in dominant IRD, specifically in individuals of European ancestry, and highlight a novel target for therapy.

## Methods

### Patients

Index patients and (affected) family members were recruited from Belgian ophthalmology clinics and centers for medical genetics. The study was conducted in accordance with the principles of the Declaration of Helsinki and was approved by the Ethics Committee of Ghent University Hospital (B6702021000312). All included patients consented to the study.

### Clinical investigations

The in-house p.(E519K) cohort, for which clinical investigations are described, consists of 65 patients, who have been examined at least once at the Department of Ophthalmology at Ghent University Hospital, Ghent, Belgium, the national referral center for ophthalmic genetics. Prior clinical records were revisited for general and ophthalmologic history, and for ophthalmological data recorded at first and follow-up visits. Ophthalmological data included best-corrected visual acuity (BCVA), ISCEV-standard full-field flash electroretinography in 50% of patients and electro-oculography in five cases (Roland Consult, Brandenburg an der Havel, Germany), anterior segment and dilated fundus examination supplemented with multimodal fundus imaging including spectral-domain optical coherence tomography (OCT), long-wavelength (690 nm) reflectance and short-wavelength (588 nm) autofluorescence (SW-AF) imaging with confocal scanning-laser ophthalmoscopy (Spectralis, Heidelberg Engineering GmbH, Heidelberg, Germany), color fundus photography (Clarus 700, ZEISS, Jena, Germany; and/or TRC-IX50 and TRC-DX50, Topcon Corporation, Tokyo, Japan).

BCVAs were measured using logMAR charts and are expressed in decimal equivalents. Values above 1.00 were limited to 1.00 as testing was not consistently continued beyond this point. Off-chart values were equalized with decimal values as previously described^18^. Five eyes of three patients were excluded for clinical analysis due to comorbidity with a more severe IRD or retinal trauma bringing the total to 125 eyes of 63 cases for clinical analysis. Of these, an additional five eyes (three cases) had their symptomatology and BCVAs excluded due to a comorbidity expected to affect these variables (Table 1). These factors are the reason the total number of cases (denominators) are not the same for every ratio presented.

### Short-read whole genome sequencing (srWGS)

Short-read whole genome sequencing (srWGS) was performed for patients F5-III:2, F5-IV:3 and F8-II:4. Library preparation and sequencing were performed using the Illumina DNA polymerase chain reaction (PCR)-Free LibraryPrep kit and 150 base pairs (bp) paired-end sequencing (S4, 300 cycles, NovaSeq 6000, Illumina, CA, USA). Data were basecalled using bcl2fastq2 (v2.20)^19^ and basecalled reads were subsequently aligned to GRCh38 using BWA-MEM (v0.7.17)^20^. Variant calling for single nucleotide variants (SNVs) was done using HaplotypeCaller from the GATK (v3.3)^21^ toolkit and annotation was performed through Ensembl Variant Effect Predictor (v110.0)^22^ and dbNSFP (v4)^23^. Post-demultiplexing steps were performed within the bcbio toolkit^24^. Resulting SNV VCF files were analyzed using the in-house tool Seqplorer (https://seqplorer.cmgg.be/user/login/) for coding variants in the RetNet panel (v7, 324 genes) and the online platform Franklin (Genoox, https://franklin.genoox.com/) for coding and non-coding variants in the same panel. Structural variants (SVs) overlapping with this gene panel were assessed using Manta (v1.6.0)^25^ and Delly (v0.8.7)^26^. Variants were classified using American College of Medical Genetics and Association for Clinical Genomic Science guidelines with adaptations^27^. The strength of some criteria was altered under certain conditions as recommended in literature^27–31^.

### Whole exome sequencing (WES)

A subset of index patients underwent WES, revealing p.(E519K) (Table 1, Supplementary Table 1). In addition, targeted sequencing of in-house index patients (F12-II:1, F13-II:2, S08, S09, S11, S13, S14, S17, S18 and S21) was supplemented with WES to exclude the presence of additional (likely) pathogenic variants in IRD genes. Library preparation and sequencing were performed using the SureSelectXT Human All Exon V6 or V7 (Agilent, CA, USA), KAPA HyperExome V1 (Roche) kit or Haloplex target enrichment System (Agilent Technologies Inc., CA, USA) and 150 bp paired-end sequencing (HiSeq 3000 or NovaSeq 6000, Illumina, CA, USA). Basecalling, demultiplexing, alignment, variant calling and annotation was performed as described for srWGS data, using a bed file to restrict the analysis to coding regions. Resulting SNVs were filtered using the in-house WES analysis tool Seqplorer for the customized RetNet panel (v7). Copy number variations in these genes were assessed using ExomeDepth (v1.1.16)^32^.

An NGS-based assay, containing 351 IRD genes, was performed for patients F16-II:1, F16-II:2, S32 and S33 using the Blueprint Genetics Retinal Dystrophy Panel^33^, while patients S29 and S30 underwent sequencing of the Inherited Retinal Disorders Panel by Invitae, containing 330 genes^34^.

Patient S34 underwent high-throughput sequencing of a panel of 226 IRD genes. Coding exons and their flanking intronic regions were captured using the HaloPlex target enrichment System (Agilent Technologies Inc., CA, USA). DNA libraries were sequenced on a NovaSeq sequencer (Illumina). Data analysis was done using an in-house developed pipeline compiling the data obtained from SeqNext (JSI Medical System, Ettenheim, Germany) and GATK software. Copy number variants (CNVs) detection was performed by a quantitative analysis based on the amplicons read depth as previously described^35^.

The DNA of patient S35 was enriched using Agilent SureSelect DNA, SureSelect OneSeq 300kb CNV Backbone and Human All Exon V7 capture, followed by paired-end sequencing (Illumina). Data were demultiplexed with bcl2fastq and reads were mapped using BWA-MEM. Variant calling was performed by the GATK HaplotypeCaller, followed by filtering and annotation with Alissa Interpret software and classification with Alamut Visual. CNV detection was done via the BAM multiscale reference method using depth of coverage analysis and dynamical bins in NexusClinical, which was also used to filter and annotate the detected CNVs.

### Targeted sequencing and segregation analysis

A subset of index patients (Table 1, Supplementary table 1) that had previously tested negative for mitochondrial retinopathy, or for *ABCA4*, *BEST1* or *PRPH2* targeted testing, underwent targeted sequencing for p.(E519K). Fifty-one family members underwent segregation analysis for p.(E519K). PCRs were performed using Kapa2G Robust Master Mix (2x, Kapa Biosystems, MA, USA), followed by Sanger sequencing using the BrilliantDye^™^ kit (v3.1, NimaGen, Nijmegen, Netherlands) and run on an ABI3730XL DNA analyzer (Applied Biosystems, MA, USA). Primers used for PCR and sequencing are listed in Supplementary Table 2.

### Long-read whole genome sequencing (lrWGS)

Long-read whole genome sequencing (lrWGS) was executed for patients F5-III:2, F5-IV:3, F8-II:3; and F8-II:4. Library preparation was performed using the SQK-LSK114 ligation sequencing kit (Oxford Nanopore Technologies (ONT), United Kingdom), followed by sequencing on a PromethION 24 system (ONT), using an individual FLO-PRO114M (ONT) flow cell for each sample, for a maximum of 72 hours (h). Basecalling was done through Guppy (v.6.4.6, ONT) after which reads were filtered on a minimum length of 100 bases and a minimum average read quality score of 10. Reads were trimmed and mapped to GRCh38.p14 via minimap2 (v2.24)^36^. SVs were called with SVIM (v1.4.2)^37^, Sniffels2 (v2.0.7)^38^, and CuteSV (v2.0.2)^39^ after which a consensus VCF was generated using Jasmine (v1.1.5)^40^. SNVs were called using Clair3 (v1.0.5)^41^ and phased using WhatsHap (v2.0)^42^. Coverage analysis was done for all alignment files using mosdepth (v0.3.3)^43^ and quality metrics were assessed through pycoQC (v2.5.2)^44^ and NanoPlot (v1.40.0)^45^.

### Microsatellite analysis

Microsatellite markers (Supplementary Table 3) were PCR amplified with 10x PCR buffer (Invitrogen, MA, USA), 50 mM MgCl_2_ (Invitrogen), 1 mM dNTPs (New England Biolabs (NEB), MA, USA), DMSO (Sigma-Aldrich, MO, USA), 5 u/μl Taq (Invitrogen), and 10 μM primer. One μl of product was mixed with 0.3 μl ROX-500 size standard (GeneScan, Freiburg im Breisgau, Germany) and 10 μl Hi-Di formamide (Applied Biosystems). PCR fragments were size fractionated on an ABI3730XL capillary sequencer and data were analyzed using GeneMapper software (v5, Applied Biosystems).

### Generation of expression vectors and site-directed mutagenesis

Human *RPE65* and *CRALBP* cDNAs, obtained from gBlocks (IDT, IA, USA), were subcloned into bicistronic pVitro2 expression vector (InvivoGen, CA, USA). An expression vector containing *RPE65* cDNA in the pcDNA3.4 backbone was obtained from GenScript (New Jersey, USA). The p.(D477G) and p.(E519K) variants were introduced in the *RPE65* sequence in both vectors, and HA- and MYC-tags were introduced into the *RPE65*-pcDNA3.4 vector, using the Q5 Site-Directed Mutagenesis Kit (NEB). Primers used to amplify the gBlocks, mutagenesis PCR and PCR to verify the mutagenesis are listed in Supplementary Table 4.

### Cell culture and transient transfection

HEK 293-T cells (ATCC, Virginia, USA) were maintained in Dulbecco’s Modified Eagle Medium (DMEM, Gibco, Thermo Fisher, MA, USA) supplemented with 10% fetal bovine serum (FBS, Greiner Bio-One, Kremsmünster, Austria) and 1% Penicillin-Streptomycin (Gibco) at 37 °C with 5% CO_2_. Adult Retinal Pigment Epithelium-19 (ARPE-19, ATCC) cells were maintained in DMEM-F12 (Gibco), supplemented with 10% FBS, 1% Penicillin-Streptomycin, 1% Non-essential amino acids (Gibco), 1% Glutamax supplement (Gibco) and 0.1% Amphotericin B (Gibco). Cells were seeded, 0.75 – 3 × 10^4^ cells (12-well plate) and 2.2 × 10^6^ cells (10 cm culture dish) and transfected using Lipofectamine 3000 transfection agent (Invitrogen) or TransIT-X2 (Mirus Bio, WI, USA) with 1 mg/ml – 1 μg/ml vector, depending on the experiment. Negative control samples were transfected without vector.

For the *in vitro* isomerase activity assay, pVitro2 expression plasmids containing either WT-RPE65 or p.(E519K)-RPE65 were prepared by using QIAprep Maxi kits (Qiagen, CA, USA). Next, 30 μg of pVitro2 plasmid (WT or mutant) were transfected into human Expi293 suspension cells (Thermo Fisher Scientific) using 40 μl of ExpiFectamine transfection reagent (Thermo Fisher Scientific). The cells were incubated on an orbital shaker at 37 °C and 8% CO_2_ for 16–18 h. At 18 h post transfection Expifectamine 293 Enhancer 1 (150 mL/flask) and Expifectamine 293 Enhancer 2 (1.5 mL per flask) were added. All-*trans* retinol was added 42 h post-initial transfection, at a final concentration of 2.5 μM. Finally, cells were harvested at 6 h after substrate addition (total 48 h transfection to harvest) for downstream retinoid analysis.

### Retinoid extraction, saponification, and retinoid analysis

All procedures were performed under red safelights. For analysis of WT and p.(E519K)-RPE65 activity in Expi293 cells, retinoids were extracted and saponified as described^46^ from cells harvested by centrifugation from 30 ml volumes of cultured transfected Expi293 cells. Isomeric retinols were analyzed on 5 *μ*m particle LiChrospher Si-60 (Alltech, IL, USA) normal phase columns (2 × 250 mm) on an Agilent 1100/1200 series HPLC system (Agilent Technologies, DE, USA), in hexane mobile phase containing ethyl acetate (11.2%):dioxane (2.0%):octanol (1.4%), following Landers & Olson, 1988^47^, as earlier modified^46^. Data were analyzed using ChemStation32 software (Agilent).

### Western blot

Proteins were isolated using radioimmunoprecipitation assay buffer (RIPA, 94%, Sigma-Aldrich), protease inhibitor (4%, Roche, Basel, Switzerland), phosphatase inhibitor 2 (1%, Sigma-Aldrich), and phosphatase inhibitor 3 (1%, Sigma-Aldrich). Assessment of protein yields was performed according to the Pierce BCA protein assay protocol (Thermo Fisher). Total protein (15–25 μg) was separated by electrophoresis on Nu-Page 4–12% Bis-Tris Gel (Invitrogen) under reducing conditions and transferred to a nitrocellulose membrane (Invitrogen). Membranes were blocked with 5% membrane blocking agent (Cytiva, MA, USA) and incubated with either anti-RPE65 antibody (1:600, 17939–1-AP, Proteintech Europe, Manchester, UK), anti-CRALBP (1:4000, ab1501, Abcam, Cambridge, UK), or anti-ß-tubulin (1:2500, ab6046, Abcam) 1.5 – 2 h at room temperature. Membranes were washed with 1x Tris-buffered saline with 0.5% Tween20 and incubated with secondary anti-rabbit or mouse antibody (1:2500, Cell Signaling Technology, MA, USA). Membranes were developed with SuperSignal West Dura Extended Duration Substrate (Thermo Fisher). Expression levels of CRALBP were used to evaluate transfection efficiency and to normalize RPE65 expression. Quantification of RPE65 and CRALBP expression was performed using the ImageLab software (v6.1, Bio-Rad, CA, USA).

### Cellular thermal shift assay (CETSA)

Ten million HEK 293-T cells were transfected (single or co-transfected) with 1 mg/ml WT-RPE65, p.(D477G) and/or p.(E519K) expression vector as described above. Forty-eight h after transfection, the cells were pelleted, dissolved in 1 ml 1x PBS (Gibco) and distributed over 12 tubes and spun down. Eighty μl of PBS was removed and each tube was incubated at a specific temperature within the following range 30 – 66.9 °C. Proteins were extracted by repeated freeze-thaw cycles using liquid nitrogen, followed by addition of 80 μl 1x PBS and ultracentrifugation (Optima TLX ultracentrifuge, Beckman Coulter, CA, USA). The supernatant was transferred, and protein concentrations were determined by the Pierce BCA protein assay kit (Thermo Fisher). Western blot was performed using anti-RPE65 antibody as described above.

### Minigene assays

To test the effect of *RPE65* variants p.(E519K) and p.(D477G) on splicing *in vitro* splice assays were used. A 3.4 kb genomic segment of *RPE65* spanning exons 11–14 was amplified from human genomic control DNA (Roche). The WT-*RPE65* PCR product was restricted and subsequently subcloned into the pSPL3_2096 vector, a derivative of the exon-trapping vector pSPL3 (Invitrogen-Life Technologies, Carlsbad, CA, USA), containing two HIV-TAT exons (TAT1, TAT2), flanking the insert. Mutant constructs were obtained using the Q5 Site-Directed Mutagenesis kit (NEB, MA, USA). Integrity of constructs was assessed using ONT long-read sequencing (Plasmidsaurus, Eugene, OR, USA). Constructs were transfected in HEK293T cells using Lipofectamine 3000 (Invitrogen). After 24 h, total RNA was extracted (RNeasy Mini Kit, Qiagen Benelux, Antwerp, Belgium) and used as input to obtain cDNA (iScript Select cDNA synthesis kit, Bio-Rad Laboratories). cDNA was PCR amplified with pSPL3 exon primers and separated via agarose gel (2% TBE) electrophoresis, followed by Sanger sequencing (Applied Biosystems), next generation sequencing (NGS, MiSeq, Illumina) and ONT long-read sequencing (Eurofins Genomics, Germany). Primers used for cDNA synthesis and cDNA PCR are listed in Supplementary Table 5.

To calculate exon ratios NGS and ONT data was aligned against the corresponding construct sequence using STAR (v2.7)^48^ and minimap2 (v2.24)^36^ respectively. Coverage for each position of the construct was determined using samtools depth (v1.18)^49^, and mean coverage was calculated for exons included in the construct. Cumulative bar graphs for the relative coverage for each region were made with ggplot2 (v3.4.3) in RStudio (v2023.12.1+402).

### Co-immunoprecipitation (Co-IP)

Plasmids expressing HA-tagged WT-RPE65 and MYC-tagged p.(D477G) or p.(E519K)-RPE65 were either individually or co-transfected at an equal molar ratio in ARPE-19 cells, using TransIT-X2 (Mirus Bio, WI, USA) at 1 mg/ml and a 3:1 TransIT-X2/DNA ratio. At 48 h post transfection, total cellular proteins were extracted as described above. Co-IP was performed with protein A Dynabeads following manufacturer’s protocol (Invitrogen, MA, USA) using the anti-MYC (2 μg, ab9106, Abcam), the anti-HA (2 μg, ab9110, Abcam) and the anti-IGG antibodies (4 μg, ab2410, Abcam). Western blot was performed as mentioned earlier with the abovementioned anti-MYC (1:2000) and anti-HA (1:4000) antibodies.

### RPE65 protein crystal structure and modeling with AlphaFold

All monomer protein structures were modeled using the standalone version of AlphaFold (v2.0.0)^50^. WT RPE65 was modeled using a fasta file containing the UniProt sequence (https://www.uniprot.org/uniprotkb/Q16518/entry#sequences) as the only input for the ‘–fasta_paths’ flag. For missense isoforms modeling, this sequence was adapted to the predicted amino acid sequence resulting from each respective missense variant. Dimer structure predictions were modeled using AlphaFold (v2.3.1) with the ‘multimer’ model preset. Fasta files, used as the only input for the ‘–fasta_paths’ flag, contained only the two respective monomer structure sequences for each dimer structure prediction of interest. The ranked0 structures and crystal structures, 3FSN^51^ and 4RYX^52^ were visualized using UCSF ChimeraX (v1.7.1, RBVI with support of the National Institutes of Health, University of California, CA, USA). ChimeraX was also used to perform an electrostatic surface analysis.

### *In silico* predictions

*In silico* (splice) prediction scores were obtained for NM_000329.3: c.1430A>G (chr1: g.68431085T>C) and NM_000329.3: c.1555G>A (chr1:g.68429823C>T) using dbNSFP (v4.7)^53^ , PolyPhen-2^54^, MutationTaster2021^55^, Mutscore^56^, AlphaMissense^57^, SpliceAI lookup (Max distance 5000)^58^ and Aggrescan^59^.

### Statistical analysis

Data visualization and statistical analyses were performed with GraphPad Prism v10.2.0 for macOS (GraphPad Software, MA, USA). A Kruskal-Wallis test with Dunn’s multiple comparisons test was used to compare protein expression of Western blot, while a non-linear regression was used to generate the melting curves for CETSA.

## Results

### A novel *RPE65*-related dominant retinopathy caused by monoallelic variant p.(E519K)

**Figure 1.**
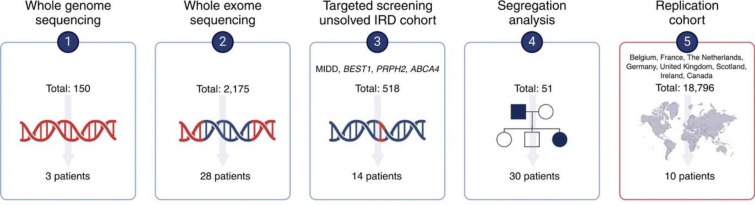
Workflow through which monoallelic patients for RPE65 c.1555G>A p.(E519K) were identified. (1–4) represent the Belgian IRD discovery cohort: WGS analysis (n=150) and a second step of WES analysis (n=2,175) in IRD cases revealed c.1555G>A, p.(E519K) in 3 and 28 cases, respectively. Targeted testing of IRD patient cohorts that tested negative for mitochondrial retinopathies (MIDD), ABCA4-, BEST1-, and PRPH2-associated maculopathy (n=518) identified 14 more patients. Segregation analysis (n=51) showed segregation of p.(E519K) with disease in 30 affected family members. The total number of monoallelic p.(E519K) IRD cases in Belgian discovery cohort: n=75. (5) represents the IRD replication cohort (n=18,796). p.(E519K) was mined in IRD registries from France, The Netherlands, Germany, United Kingdom (UK), Scotland, Ireland, Canada. The total number of monoallelic p.(E519K) IRD cases found in the replication cohort: n=8, specifically France (n=3; of which one included in this study), the Netherlands (n=1), Canada (n=6). Overall number of monoallelic p.(E519K) IRD cases: n=85, of which 83 are included in this study.

WGS was performed in three WES-prescreened patients (F5-III:2, F5-IV:3 and F8-II:4) with AD IRD hallmarked by macular involvement. Prioritization of variants in known IRD genes uncovered a shared, monoallelic *RPE65* variant, c.1555G>A, p.(E519K), initially classified as a variant of unknown significance (VUS). The variant is present in gnomAD v2.1.1 (rs373274945) with an allele frequency of 0.0004014 (allele count: 1, no homozygotes). Subsequently, in-house WES-data from 2,175 individuals with IRD was queried, identifying 28 additional monoallelic p.(E519K) patients (Fig 1).

As their phenotypes often resembled those seen in mitochondrial maculopathy or *ABCA4*-, *BEST1*-, and *PRPH2*-associated maculopathies, an IRD cohort that previously underwent targeted testing for these indications was screened, expanding the p.(E519K) cohort with 14 additional patients. The in-house index patients, identified using this targeted approach, underwent WES to exclude additional (likely) pathogenic variants in IRD genes. Performed genetic analyses and identified variants are listed in Supplementary Table 1. Subsequent segregation analysis revealed 30 clinically affected family members heterozygous for p.(E519K), confirming the dominant inheritance pattern initially established through pedigree analysis (Supplementary Fig 1). Taken together, the Belgian IRD discovery cohort contains 75 monoallelic p.(E519K) cases, all of Flemish origin (in-house cases: *n*=65; external referrals: *n*=10). Furthermore, we expanded our search to other IRD patient cohorts (France, the Netherlands, Germany, United Kingdom [UK], Scotland, Ireland, Canada), representing an IRD replication cohort of 18,796 individuals. Additional monoallelic p.(E519K) IRD cases were identified in the IRD cohorts from France (*n*=3, of which one included in this study), the Netherlands (*n*=1), and Canada (*n*=4, British Columbia; *n*=2, Alberta). To obtain prevalence data in matched controls, we also mined diagnostic non-IRD genomic databases from these regions (overview provided in Supplementary Table 6). Variant p.(E519K) was not found in any of these non-IRD genomic databases collectively representing 136,306 individuals (Supplementary Table 6).

A total of 85 monoallelic p.(E519K) individuals with IRD were identified in Belgium, France, the Netherlands, and Canada. This study includes genomic and clinical data from 83/85 p.(E519K)-IRD patients, including 48 familial cases, and 35 cases whose families were not available for genetic or clinical investigations.

### Index patients with p.(E519K) share a common haplotype of 464 kb

**Figure 2.**
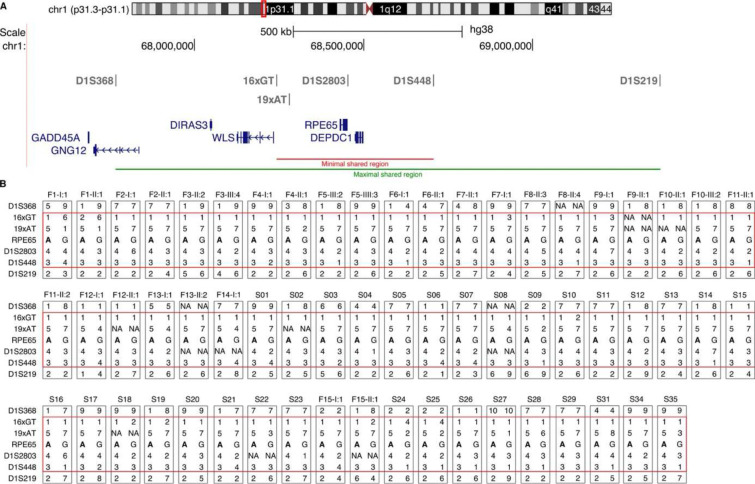
Haplotype reconstruction in all Belgian index patients, one family member (when available) and the French (S34), Dutch (S35) and Canadian (S31) index cases. **(A)** The location of the six microsatellite markers is depicted above a schematic representation of the genes in this region (chr1: 67611911–69469522). Red line: minimal shared region (464 kb), green line: maximal shared region (1.6 Mb). **(B)** Results from haplotype analysis. Each grey box represents the two alleles with the patient identifier shown at the top. The c.1555G>A (p.(E519K) variant is indicated in bold and the common allele is shown on the left. The red box highlights the shared markers. NA values: missing data. The conversion key for the microsatellite alleles can be found in Supplementary Table 9.

As the majority of IRD cases from the discovery cohort had a reported Flemish origin, a founder effect was suspected. Initial delineation of a common haplotype was performed using long-read WGS on four patients (F5-III:2, F5-IV:3, F8-II:3 and F8-II:4). Established boundaries were used for further haplotype reconstruction using six microsatellite markers flanking *RPE65* in all Belgian (in-house and external) index patients, one of their affected family members (if available), and the French (S34), Dutch (S35) and Canadian (S31) index cases (Fig 2). A minimal shared haplotype of 464 kb between all affected cases was identified, spanning *RPE65* and *DEPDC1*, while the maximal shared region of 1.6 Mb, additionally contains *WLS*, *DIRAS3* and part of *GNG12*. None of these genes, except for *RPE65*, are associated with IRD. Apart from p.(E519K), no other plausible (likely) pathogenic variants were found in the shared region.

### *In silico* and AlphaFold protein modeling supports a destabilizing effect of p.(E519K)

**Figure 3.**
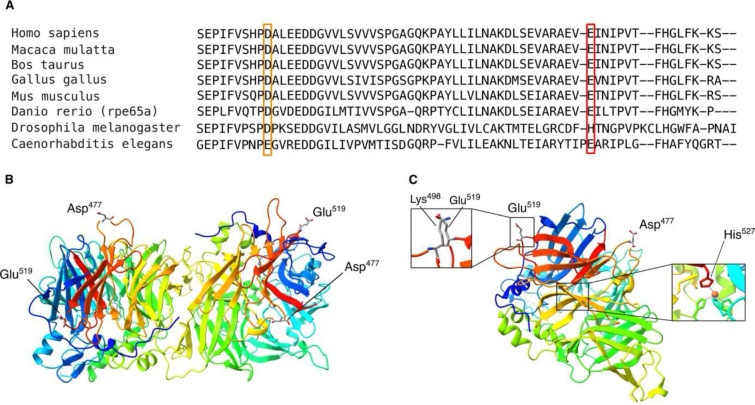
Conservation and protein modelling using crystal structures of RPE65. (**A)** The final segment of the amino acid alignment for RPE65 of six vertebrate species and the related carotenoid cleavage dioxygenases from two invertebrate species is displayed. Orange box: Asp477; red box: Glu519. **(B)** In the 3FSN crystal structure, RPE65 is observed as a dimer. Both Asp477 and Glu519 are distant from the dimerization site. **(C)** The monomer crystal structure of RPE65 (4RYX), whereby a positively charged lysine (Lys498) is positioned in the vicinity of Glu519. If the glutamate is replaced by a lysine these residues (Lys498 and Lys519) will repel each other due to their positive charges. His527, important for iron binding in the catalytic center, could be affected by p.(E519K).

The RPE65 protein is evolutionarily well-conserved, which also applies to Glu^519^ and Asp^477^ (Fig 3a, Supplementary Fig 2a). When considering their paralogous residues through the wider carotenoid cleavage dioxygenase (CCD) superfamily, of which RPE65 is a member, Glu^519^ is conserved in all mammals, and in one of the three zebrafish ohnologs.

To assess the potential impact of p.(E519K) and compare its properties with p.(D477G), 24 *in silico* predictions were evaluated. Overall, predictions for p.(E519K) compared to p.(D477G) were more damaging or deleterious (Supplementary Table 7). Moreover, Aggrescan predicts that both variants are more prone to aggregation compared to WT RPE65 (Supplementary Fig 2b-d).

Furthermore, crystal structures of RPE65 were examined and AlphaFold models were generated to study the impact of p.(E519K) and p.(D477G) on protein structure and stability. The RPE65 protein exhibits a seven-bladed ß-propeller structure, with Glu^519^ situated on blade VII and Asp^477^ on blade VI (Supplementary Fig 3). Both residues are positioned on the protein’s exterior, away from key sites such as the substrate tunnel, water tunnel and the dimerization site (Fig 3b). AlphaFold models for WT-RPE65, p.(E519K) and p.(D477G), in addition to AlphaFold-Multimer modelling, show nearly identical folding and dimer formation, suggesting a minimal impact on protein structure (Supplementary Fig 4). However, based on crystal structures of RPE65 (3FSN; 4RYX), p.(E519K) may induce protein instability due to the proximity of a positively charged Lys^498^, causing a repulsion effect with Lys^519^ (Fig 3c and Supplementary Fig 4a). Moreover, Glu^519^ is located close to His^527^ on blade VII, crucial for iron binding in the catalytic center (Fig 3c and Supplementary Fig 3). Consequently, p.(E519K) could indirectly influence enzymatic RPE65 activity. In addition, the electrostatic surface analysis for p.(E519K) shows a shift from a negative to a positive potential at position 519 and the surrounding area (Supplemental Fig 5).

### Variant p.(E519K) affects expression, enzymatic activity and thermal stability of RPE65

**Figure 4.**
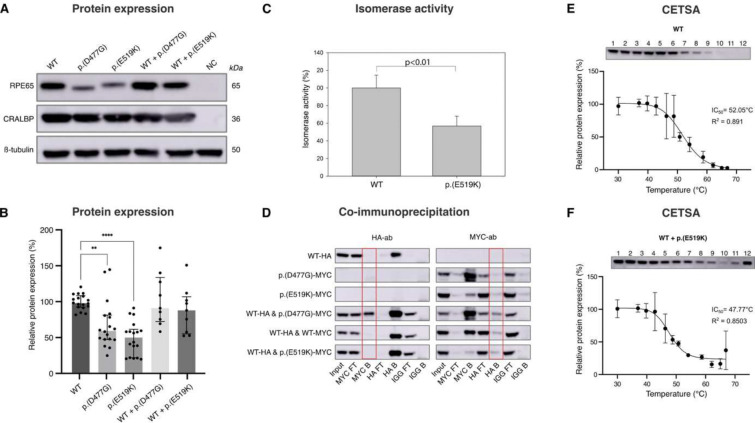
Variant p.(E519K) affects expression, enzymatic activity and thermal stability of RPE65. **(A)** RPE65 protein expression following single or co-transfection of vectors encoding WT-RPE65, p.(D477G), or p.(E519K) proteins in HEK293-T cells. A shift was observed for the p.(D477G) variant. CRALBP and ß-tubulin protein expression were used as transfection and loading controls. NTC: no template control. **(B)** Relative RPE65 protein expression, normalized using CRALBP protein expression, is shown. A significant decrease in protein expression is observed after overexpression of p.(D477G) and p.(E519K), compared to WT-RPE65. Data are represented as median ± IQR. n = 18 for WT, p.(D477G) and p.(E519K), n=9 for WT+p.(D477G) and WT+p.(E519K), * is p<0.05 according to a Kruskal-Wallis test with Dunn’s multiple comparisons test. **(C)** p.(E519K) RPE65 showed reduced retinol isomerase activity (56%) compared to WT-RPE65 when measured in an in cellulo minimal visual cycle assay. **(D)** In co-IP experiments, ARPE-19 cells were either single or co-transfected with constructs encoding HA-tagged WT-RPE65 and/or MYC-tagged p.(D477G) or p.(E519K) protein. Co-IP was performed, using the anti-HA and anti-MYC antibodies and western blotting with either anti-HA or anti-MYC antibody was used to determine coprecipitation. The results demonstrate an interaction between the D477 and WT-RPE65 proteins, but not between p.(E519K) and WT-RPE65 proteins. Input represents total protein extract, FT (flow-through) the leftover unbound fraction, and B (bound) proteins specifically bound by the antibodies and precipitated by the beads. **(E-F)** Results from a CETSA assay to assess thermal stability after transfection of WT-RPE65 **(E)** and co-transfection of WT and p.(E519K) **(F)** in HEK293-T cells. The IC50, the temperature at which 50% of the protein is denatured and aggregated, was lowered in the WT + p.(E519K) condition. Immunoblots are depicted above the graphs, labels 1–12 represent the temperatures at which the cells were incubated (30, 37, 39.8, 42.8, 46.3, 48.8, 50.8, 54.1, 58.7, 62.1, 65.0 and 66.9°C, respectively). A non-linear regression was performed on the data and resulting means with standard deviation are shown in the graphs. R^2^ quantifies goodness of fit.

SpliceAI predicts a potential splicing change for p.(D477G) but not for p.(E519K) (Supplementary Fig 6a). Minigene assays showed aberrant splicing for p.(D477G), including multiple exon skipping events with or without exon 13 skipping (Supplementary Fig 6b,c). Although usage of the strengthened cryptic acceptor site at c.1430 was noted for p.(D477G), this was not increased compared to WT or p.(E519K) (Supplementary Fig 6c). Variant p.(E519K) does not seem to induce an aberrant splicing effect in HEK293-T cells, although a relatively increased ratio of reads containing exon 14 (including 3’UTR) was noted, possibly due to strengthening of the canonical acceptor site of exon 14 (Supplementary Fig 6d). HEK293-T cells were single- or co-transfected with WT-RPE65, p.(D477G) and/or p.(E519K) constructs, followed by immunoblotting. Reduced RPE65 protein expression of p.(D477G) and p.(E519K) compared to WT-RPE65 was found at 59.3% and 49.8%, respectively (p=0.0016 and p>0.0001), following single transfection (Fig 4a,b). Co-expression of WT/p.(D477G) and WT/p.(E519K) did not result in any significant reduction of protein expression (p>0.9999 and p=0.7605) (Fig 4a,b).

Migration of the p.(E519K) protein resembles that of the WT protein, unlike p.(D477G), migrating marginally faster, as previously shown^12^. In addition, we found that p.(E519K)-transfected Expi293 cells produced about 56% of the 11-*cis* retinol compared to WT-RPE65 (p<0.01) (Fig 4c). Using a CETSA assay the inhibitory concentration (IC_50_) temperature, whereby 50% of the RPE65 protein is denatured and aggregated, showed a difference between WT, p.(D477G), p.(E519K), WT/p.(D477G) and WT/p.(E519K) (Fig 4e,f and Supplementary Fig 7). Mutant (co-)transfections resulted in an IC_50_ lower than WT (WT/p.(E519K)=47.77°C; WT/p.(D477G)=49.11°C; WT=52.05°C), suggesting p.(D477G) and p.(E519K) result in thermal instability (Fig 4f).

Following overexpression experiments in ARPE-19 cells, co-immunoprecipitation showed no (differential) interaction between WT-RPE65 and p.(E519K), in contrast to p.(D477G), which clearly interacts with WT-RPE65 (Fig 4d).

### Comprehensive phenotyping reveals two distinct and recognizable p.(E519K)-IRD phenotypes

An in-depth clinical characterization was performed on 63 in-house patients with AD p.(E519K)-IRD (F1-F14, Supplementary Fig 1). Thirty patients (50.0%) complained of reduced visual acuity and central scotomata (median age at onset 57 years; range 24–76 years) (Table 1 and Supplementary Table 8). The other 30 (50.0%) did not self-report subjective symptoms but showed clinical signs upon ophthalmological investigation (median age at diagnosis 53 years; range 18–74 years) (Table 1 and Supplementary Table 8). BCVA at last exam ranged from 0.01 to 1.00 (median=0.65, interquartile range (IQR)=0.55) in the symptomatic group, and from 0.70 to 1.00 (median=1.00, IQR=0.10) in the asymptomatic group (Supplementary Table 8). Full-field electroretinography in 30 (47.6%) showed predominant rod-system involvement in 14 (46.7%) (Supplementary Table 8).

**Figure 5.**
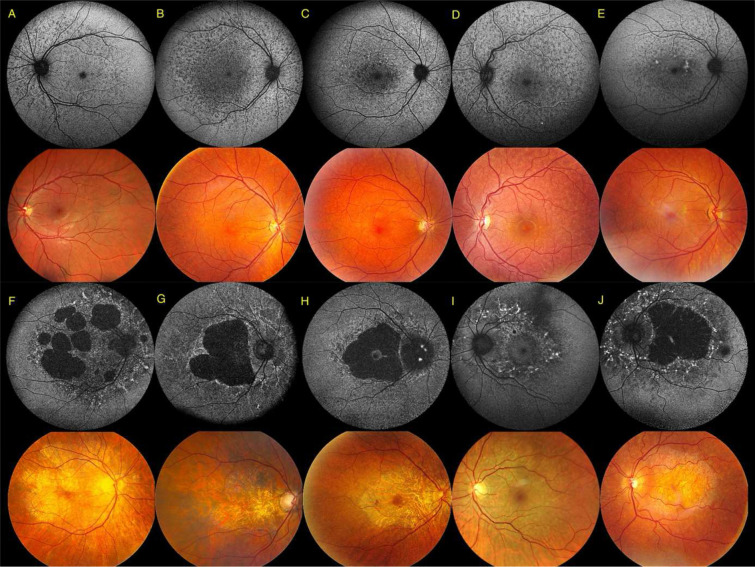
SW-AF imaging (top) and color fundus imaging (bottom) illustrating the representative distinct p.(E519K)-associated phenotypes: mottled phenotype (A-E) and pattern dystrophy (F-J). **(A)** Left eye of 31-year-old female (F12-II:1). **(B)** Right eye of 41-year-old male (S02). **(C)** Right eye of 43-year-old male (F11-II:2). **(D)** Left eye of 29-year-old female (F2-III:1). **(E)** Right eye of 47-year-old female (F11-II:1). **(F)** Right eye of 59-year-old female (S10). **(G)** Right eye of 78-year-old male (F4-I:1). **(H)** Right eye of 72-year-old female (F3-II:2). **(I)** Left eye of 62-year-old female (F5-III:7). **(J)** Left eye of 58-year-old male (F8-II:5). SW-AF = short-wavelength autofluorescence.

SW-AF imaging allowed categorization into two distinct phenotypes. A first subgroup of 27 cases (43.5%) was referred to as a ‘mottled phenotype’ (Fig 5a–e) because of small, diffuse, hypo-autofluorescent (hypo-AF) spots spread over the posterior pole. These usually extended beyond the vascular arcades and the optic nerve head up to the retinal midperiphery. This hypo-AF mottling was often associated with variable hyperautofluorescent (hyper-AF) lesions. The second subgroup was classified as a ‘pattern dystrophy’, observed in 35 cases (56.5%) and is characterized by more organized hyper-AF changes as predominant feature (Fig 5f–j). Macular chorioretinal atrophic lesions were seen in 55 eyes (44.0%) and associated in all but one eye (S13), with pattern dystrophy. Chorioretinal atrophy was either bilateral (22; 68.8%) or unilateral (10; 31.3%) (Supplementary Table 8). Although an extensive variability was noted in the p.(E519K) cohort (Supplemental Fig 10), lower visual acuities and larger surface areas of chorioretinal atrophy tended to be associated with older age.

Color fundus images often showed subtle changes compared to SW-AF imaging (Fig 5). Unifocal vitelliform lesions were observed in 11 eyes (8.8%), at the fovea (Supplementary Fig 11). Electro-oculography in four of these six cases showed two cases with mildly subnormal light peak-to-dark trough ratios (1.7–1.8). Cystoid macular edema was observed in 15 eyes (12.0%), seven of which had a vitelliform lesion. Structural retinal layer abnormalities were primarily located at the three outermost hyperreflective bands on OCT. The innermost of these, the ellipsoid zone (EZ), often showed a fine sinusoidal deformation. More severe EZ alterations were observed, often in older eyes. Lesions hyper-AF on SW-AF imaging appeared as hyperreflective, tall, smooth, bell-shaped lesions or as wider-than-tall lesions with an irregular surface arising from the hyperreflective band corresponding to the RPE-Bruch membrane complex. Chorioretinal atrophic lesions often showed degenerative intraretinal cavitation and outer retinal tubulations (Supplementary Fig 12). The phenotypes observed in patients referred by external experts (S22-S35, F15-I:1, F15-II:1, F16-II:1, F16-II:2) are indistinguishable from those seen in the in-house IRD cohort (Supplementary Fig 13). Several particular phenotypes were found in our overall p.(E519K)-IRD cohort. Two patients (F10-II:1 and S20) had been considered molecularly solved due to biallelic pathogenic *ZNF408* and *CEP290* variants. Their severe early-onset IRD had masked the p.(E519K)-IRD (Supplementary Figures 8 and 9). For this reason, these patients were excluded from the aforementioned clinical analyses. Another outlier is S22, displaying a retinitis pigmentosa-type rod-cone dystrophy (RCD). No other coding (likely) pathogenic variants that could explain the RCD were identified using WES (Supplementary Fig 14).

## Discussion

We present and characterize *RPE65* variant p.(E519K) as a novel cause of dominant IRD, often manifesting as an adult-onset maculopathy. The common haplotype of 464 kb was shared by all monoallelic cases of the discovery cohort and by the index cases of the replication cohort, supporting a founder effect. The geographical background of the Belgian cases suggests a Flemish origin of p.(E519K). Hence, the presence of p.(E519K) in IRD patients from regions where Flemish people migrated to, specifically France, the Netherlands and Canada, is not surprising. Its overall rarity in gnomAD v2.1.1 as well as in the other genomic databases we mined emphasizes the importance of increasing genetic ancestral diversity in population-scale datasets^60^.

The fact that another *RPE65* variant has been reported at the same location, c.1555G>T, p.(Glu519Ter), in the context of AR *RPE65*-IRD, suggests this guanine is prone to variation^61^. *In silico* predictions and (dimer) protein modelling support a deleterious effect of p.(E519K) even stronger than for p.(D477G). Protein instability of p.(E519K) can be explained by a repulsion effect between Lys^519^ and Lys^498^, the shift in surface potential, as well as a potential destabilising effect on His^527^, essential for iron binding in the catalytic centre. Moreover, a significantly reduced protein expression as well as reduced isomerase enzymatic activity of p.(E519K) compared to WT was demonstrated, similar to p.(D477G)^16,17^. Pure loss-of-function (LOF) is unlikely to be the only effect, as heterozygous carriers of *RPE65* LOF variants exhibit no phenotype. However, a larger cell type-specific LOF effect might not be captured *in vitro*. Moreover, both the thermal instability induced by p.(E519K), together with an increased aggregation hot spot, support that p.(E519K) is prone to aggregation, a known effect of p.(D477G) and of variants in other IRD genes^17,62,63^. While we confirmed the known interaction between p.(D477G) and WT-RPE65^17^, no interaction was observed between p.(E519K) and WT-RPE65, making a dominant-negative effect in this cell type less likely. Although aberrant splicing had been demonstrated for p.(D477G)^16^, we could not demonstrate a strong effect on splicing for p.(E519K), also supported by SpliceAI. However, a cell type-specific splicing effect in patient-derived RPE cells cannot be excluded, as splicing is highly cell-type specific ^15–17,64–68^.

Several knock-in p.(D477G) mice (both WT/KI and KI/KI) have been generated, of which the phenotypes were highly variable and dependent on age, luminescence exposure and genetic background^15,16,65,66,69^. These mice lacked a discernible phenotype and thus did not accurately recapitulate the human situation. More biologically relevant models, such as patient-derived induced pluripotent stem cell (iPSC)-retinal pigment epithelium (RPE) (iPSC-RPE), mimic the native conditions of RPE65, including its native splicing, expression, localisation and interactions patterns, and will contribute to understanding the pathomechanisms of p.(E519K)- and p.(D477G)-IRD.

In addition, an extensive clinical characterization of the 63 Belgian p.(E519K)-IRD patients revealed two distinct phenotypic subtypes. The hypo-AF mottled subtype (43.5%) is characterized by small, diffuse, hypo-AF spots spread out across the posterior pole. This phenotype seems pathognomonic for this novel AD *RPE65*-IRD and could guide towards its molecular diagnosis. A second predominant subtype (56.5%) displays a pattern dystrophy most often resembling the mitochondrial retinopathy due to *MT-TL1* m.3243A>G^70^. We hypothesize that the mottled phenotype might evolve into the more severe pattern dystrophy over time, representing the milder and more severe end of the p.(E519K)-IRD phenotypic spectrum, respectively. Long-term follow-up of affected individuals will be useful to support this. Lower visual acuity and larger surface area of chorioretinal atrophy were associated with older age, while variability in disease severity was observed in the patient cohort (Supplementary Fig 10). Overall, this differs from the severe rod-cone dystrophy associated with AR *RPE65*-IRD^6,71,72^ and the p.(D477G)-IRD that was initially described as an adult-onset choroideremia-like IRD with reduced or non-penetrance^12^.

Familial co-segregation analysis showed that all cases that are monoallelic for p.(E519K) displayed clinical signs upon retinal evaluation, albeit sometimes subtle and often not associated with subjective visual symptoms.

In the differential diagnosis of the p.(E519K) pattern dystrophy phenotype, mitochondrial retinopathies, *ABCA4*-IRD and *PRPH2*-IRD can be considered. Multiple atrophic lesions in patients above 50 years could be misdiagnosed as geographic atrophy in dry age-related macular degeneration (AMD). However, the additional pattern dystrophy on SW-AF imaging is different from AMD. For those patients in whom vitelliform lesions are predominant, other AD IRD caused by monoallelic variants in *BEST1*, *IMPG1*, *IMPG2* and *PRPH2* need to be considered.

Given the approved *RPE65* gene augmentation therapy *Voretigene neparvovec-rzyl* (Luxturna^®^) for patients with AR *RPE65*-IRD, unravelling the pathomechanisms of AD *RPE65*-IRD is thus of utmost importance in view of potential treatment options. For AD p.(D477G)-IRD gene augmentation therapy has been performed in a p.(D477G) KI murine model, resulting in improved retinal function^69^. Moreover, oral 9-*cis* retinyl acetate therapy has been administered to five p.(D477G) patients, three of whom displayed improved visual function^69,73^. However, the long-term effects of either of these approaches on mutant toxicity and retinal degeneration are not yet known. Gene augmentation therapy would only prove beneficial when the mechanism of action includes LOF including haploinsufficiency. As mentioned, these mechanisms alone are unlikely to cause AD *RPE65*-IRD as carriers of recessive LOF variants show no clinical signs. A gain-of-function or dominant-negative effect combined or not with a LOF seem more plausible pathomechanisms. In such scenarios, augmentation therapy could induce toxic effects^74,75^. A potential therapy could involve antisense oligonucleotides (ASOs) to specifically target and degrade the mutant allele. Proof-of-principle studies using ASOs have been performed for several IRD genes, for example *RHO*^76^. Gene editing, including CRISPR/Cas9-based or prime editing^77,78^, is another therapeutic option for this variant. This would allow to permanently correct the mutation, illustrated by EDIT-101, targeting the *CEP290* IVS26 variant (NCT03872479) ^79,80^. As a gene therapy approach for a dominant IRD is guided by its underlying mechanism, further research into its pathogenesis is needed.

Comprehensive genetic testing is key to establishing an early diagnosis in suspected p.(E519K)-IRD, which is crucial to provide adequate counselling and will only gain importance as therapies for this and similar retinal disorders will become available.

## Conclusions

Our study provides strong genetic, clinical, molecular and functional evidence for a novel, adult-onset dominant *RPE65*-associated retinopathy due to founder variant p.(E519K). These findings substantially expand the spectrum of *RPE65*-IRD, reduce the diagnostic gap in dominant IRD, and open potential new therapeutic perspectives for AD *RPE65*-IRD.

## Figures and Tables

**Table 1.** Demographics, phenotype, genetic testing method used to identify the c.1555G>A p.(E519K) RPE65 variant and optional remarks for each patient.

ID	Sex	Age at last exam (y)	Age at onset/diagnosis (y)	Phenotype	Genetic testing	Remarks
F1-I:1	M	61	61	Pattern Dystrophy	Targeted sequencing (segregation)	
F1-II:1	F	29	18	Mottled Phenotype	WES RetNet	
F2-I:1	M	90	90	Pattern Dystrophy	Targeted sequencing (segregation)	Left eye excluded due to trauma; right eye BCVA excluded due to cognitive problems
F2-II:1	F	63	63	Mottled Phenotype	Targeted sequencing (segregation)	
F2-III:1	F	34	18	Mottled Phenotype	WES RetNet	
F3-II:2	F	75	72	Pattern Dystrophy	WES RetNet	
F3-II:4	M	72	72	Pattern Dystrophy	Targeted sequencing (segregation)	
F3-III:4	F	48	47	Mottled Phenotype	Targeted sequencing (segregation)	
F3-III:6	F	45	45	Mottled Phenotype	Targeted sequencing (segregation)	
F3-III:8	F	38	38	Mottled Phenotype	Targeted sequencing (segregation)	
F4-I:1	M	79	72	Pattern Dystrophy	WES RetNet	
F4-II:1	M	58	58	Pattern Dystrophy	Targeted sequencing (segregation)	
F4-III:1	F	18	18	Mottled Phenotype	Targeted sequencing (segregation)	
F5-III:1	F	75	74	Pattern Dystrophy	Targeted sequencing (segregation)	
F5-III:2	M	69	60	Pattern Dystrophy	WGS RetNet	
F5-III:4	F	70	65	Pattern Dystrophy	Targeted sequencing (segregation)	
F5-III:5	F	68	64	Pattern Dystrophy	Targeted sequencing (segregation)	
F5-III:6	F	67	63	Mottled Phenotype	Targeted sequencing (segregation)	
F5-III:7	F	62	58	Pattern Dystrophy	Targeted sequencing (segregation)	
F5-III:8	F	60	56	Mottled Phenotype	Targeted sequencing (segregation)	
F5-IV:3	F	41	41	Pattern Dystrophy	WGS RetNet	
F6-I:1	M	68	33	Mottled Phenotype	Targeted sequencing (segregation)	
F6-II:1	F	41	29	Mottled Phenotype	WES RetNet	
F7-I:1	M	65	62	Mottled Phenotype	Targeted sequencing (segregation)	
F7-II:1	F	40	36	Mottled Phenotype	WES RetNet	
F8-II:3	M	65	58	Pattern Dystrophy	Targeted sequencing (segregation)	
F8-II:5	M	62	45	Pattern Dystrophy	WGS RetNet	
F8-III:1	F	40	40	Mottled Phenotype	Targeted sequencing (segregation)	
F8-III:2	F	37	37	Mottled Phenotype	Targeted sequencing (segregation)	
F9-I:1	M	72	72	Pattern Dystrophy	Targeted sequencing (segregation)	
F9-II:1	M	42	37	Mottled Phenotype	WES RetNet	
F10-II:1	M	68	NA	Autosomal recessive RCD	WES RetNet	IRD due to *ZNF408*: c.[16G>T];[16G>T], p.[(Glu6Ter)];[(Glu6Ter)]
F10-III:2	F	35	31	Mottled Phenotype	Targeted sequencing (segregation)	
F11-II:1	F	53	48	Mottled Phenotype	WES RetNet	Compound heterozygous carrier of two *ABCC6* variants without PXE phenotype
F11-II:2	F	46	39	Mottled Phenotype	Targeted sequencing (segregation)	BCVA excluded due to history of bilateral optic neuritis
F12-I:1	F	70	70	Mottled Phenotype	Targeted sequencing (segregation)	
F12-II:1	F	31	31	Mottled Phenotype	Targeted sequencing (specific phenotype)	
F13-I:1	M	86	70	Pattern Dystrophy	Targeted sequencing (segregation)	
F13-II:2	M	59	31	Pattern Dystrophy	Targeted sequencing (PRPH2 negative cohort)	
F13-II:4	F	55	48	Pattern Dystrophy	Targeted sequencing (segregation)	
F13-III:1	F	30	30	Mottled Phenotype	Targeted sequencing (segregation)	
F13-III:3	F	27	27	Mottled Phenotype	Targeted sequencing (segregation)	
F14-I:1	M	81	75	Pattern Dystrophy	WES RetNet	
F14-II:1	M	57	57	Pattern Dystrophy	Targeted sequencing (segregation)	
S01	M	44	24	Mottled Phenotype	WES RetNet	
S02	M	44	35	Mottled Phenotype	WES RetNet	
S03	F	49	49	Mottled Phenotype	WES RetNet	
S04	F	51	50	Pattern Dystrophy	WES RetNet	
S05	M	54	49	Pattern Dystrophy	WES RetNet	
S06	M	57	55	Pattern Dystrophy	WES RetNet	
S07	M	57	57	Pattern Dystrophy	WES RetNet	
S08	F	59	59	Pattern Dystrophy	Targeted sequencing (ABCA4 negative cohort)	
S09	F	59	54	Pattern Dystrophy	Targeted sequencing (MIDD negative cohort)	
S10	F	59	56	Pattern Dystrophy	WES RetNet	
S11	M	64	57	Pattern Dystrophy	Targeted sequencing (MIDD negative cohort)	
S12	F	66	59	Pattern Dystrophy	WES RetNet	
S13	M	67	57	Mottled Phenotype	Targeted sequencing (MIDD negative cohort)	BCVA excluded due to idiopathic congenital nystagmus
S14	M	70	70	Pattern Dystrophy	Targeted sequencing (MIDD negative cohort)	
S15	M	73	70	NA	WES RetNet	Short-wavelength autofluorescence imaging not performed
S16	M	77	76	Pattern Dystrophy	WES RetNet	Hemizygous carrier of *CACNA1F* variant without CSNB phenotype (normal ffERG)
S17	M	77	72	Pattern Dystrophy	Targeted sequencing (MIDD negative cohort)	
S18	M	78	74	Pattern Dystrophy	Targeted sequencing (MIDD negative cohort)	
S19	F	81	75	Pattern Dystrophy	WES RetNet	
S20	F	78	NA	Leber Congenital Amaurosis	WES RetNet	IRD due to *CEP290*: c.[4723A>T];[2991+1655A>G], p.[(Lys1575Ter)];[?]
S21	F	72	71	Pattern Dystrophy	Targeted sequencing (*PRPH2* negative cohort)	
S22	F	55	55	RCD/RP	WES RetNet	External patient (Belgium) Toxic alcohol/tobacco-related optic neuropathy
S23	M	65	58	Mottled Phenotype	WES RetNet	External patient (Belgium)
F15-I:1	M	82	55	Pattern Dystrophy	Targeted sequencing (segregation)	External patient (Belgium)
F15-II:1	F	60	57	Pattern Dystrophy	WES RetNet	External patient (Belgium)
S24	M	58	48	Pattern Dystrophy	WES RetNet	External patient (Belgium)
S25	M	44	39	Mottled Phenotype	Targeted sequencing (*PRPH2* & *BEST1* negative cohort)	External patient (Belgium)
S26	F	65	65	Pattern Dystrophy	Targeted sequencing (*PRPH2* & *BEST1* negative cohort)	External patient (Belgium)
S27	M	70	63	Pattern Dystrophy	Targeted sequencing (MIDD negative cohort)	External patient (Belgium)
S28	M	69	55	Pattern Dystrophy	Targeted sequencing (MIDD negative cohort)	External patient (Belgium)
S29	M	40	37	Pattern Dystrophy	WES RetNet	External patient (Belgium)
F16-II:1	F	44	33	Mottled Phenotype	Retinal Dystrophy Panel (Blueprint Genetics)	External patient (Canada)
F16-II:2	F	40	35	Mottled Phenotype	Retinal Dystrophy Panel (Blueprint Genetics)	External patient (Canada)
S30	M	57	55	Mottled Phenotype	Inherited Retinal Disorders Panel (Invitae)	External patient (Canada)
S31	F	38	35	Mottled Phenotype	Inherited Retinal Disorders Panel (Invitae)	External patient (Canada)
S32	M	72	60–70	Pattern Dystrophy	Retinal Dystrophy Panel (Blueprint Genetics)	External patient (Canada)
S33	M	65	50–60	Pattern Dystrophy	Retinal Dystrophy Panel (Blueprint Genetics)	External patient (Canada)
S34	M	24	17	Mottled Phenotype	Targeted sequencing 226 IRD genes	External patient (France)
S35	M	73	NA	Pattern Dystrophy	WES Vision disorders (505 IRD genes)	External patient (Netherlands)

External patients were seen in Belgian clinical centers other than Department of Ophthalmology at Ghent University Hospital. Abbreviations used: M = male; F = female; RCD = rod cone dystrophy; WES = whole exome sequencing; WGS = whole genome sequencing; MIDD = Maternally Inherited Diabetes and Deafness; NA = not available/applicable; PXE = Pseudoxanthoma elasticum ; CSNB = Congenital Stationary Night Blindness ; ERG = Electroretinogram.
